# Sexual size dimorphism in ground squirrels (Rodentia: Sciuridae: Marmotini) does not correlate with body size and sociality

**DOI:** 10.1186/1742-9994-10-27

**Published:** 2013-05-14

**Authors:** Jan Matějů, Lukáš Kratochvíl

**Affiliations:** 1 Departments of Zoology and Ecology, Faculty of Science, Charles University in Prague, Viničná 7 128 44, Praha 2, Czech Republic; 2 Karlovy Vary Museum, Pod Jelením skokem 30, 360 01, Karlovy Vary, Czech Republic

**Keywords:** Allometry, Constraints, *Cynomys*, *Marmota*, Phylogenetic comparative study, Social system, *Spermophilus*

## Abstract

**Introduction:**

Sexual size dimorphism (SSD) is a widespread phenomenon in animals including mammals. It has been demonstrated that across species, the direction and magnitude of sexual dimorphism in body size often corresponds to social systems. Moreover, many animal lineages conform to “Rensch’s rule”, which states that male-biased SSD increases with body size. We tested whether considerable differences in sociality and large variation in body size were connected with the evolution of SSD in the structural body size of ground squirrels, an otherwise ecologically relatively homogenous group of terrestrial rodents.

**Results:**

We found the general trend of male-biased SSD in ground squirrels, however, male size increases nearly perfectly isometrically with female size among species and sociality does not explain departures from this relationship. Species with different sociality grades significantly differ in body size, with the most social species tending to be the largest.

**Conclusions:**

We suggest that lack of conformity with Rensch´s rule in ground squirrels may be attributed to their low variation in SSD, and briefly discuss three potential causes of small magnitude of SSD in the structural size in rodents: low selection on SSD in structural dimensions, ontogenetic and genetic constraints and the existence of ecological/selection factors preventing the evolution of extensive SSD.

## Introduction

Sexual size dimorphism - a difference in size between males and females, is a widespread phenomenon in animals [1]; recently reviewed in [2]. The evolution of sexual size dimorphism (SSD) is usually ascribed to different selection pressures (natural or sexual selection) operating in males and females. Male-biased SSD is predominantly attributed to intense intrasexual competition in males [1,3], as is supported by the correlation between SSD and social or mating systems in several mammalian lineages, e.g. [4-6], as well as in mammals in general [3]. Nevertheless, the extent of SSD does not depend exclusively on male size, but it is a function of both male and female size and SSD often scales with body size. An allometric relationship between SSD and body size has been documented in a wide array of animals at various taxonomical levels, and is described by the so-called “Rensch´s rule”. This empirical rule states that male-biased SSD tends to increase with increasing body size among related species [7]. Consequently, male size increases positively allometrically with female size and males are more evolutionary plastic in body size than females [2] and references therein, [8-10]. This rule holds across the whole mammalian clade, and is followed by some, but not all, mammalian orders [3].

Although male-biased SSD generally predominates among mammals, the review of sexual dimorphism among rodents indicates that monomorphism, male-biased and female-biased SSD is typical for particular rodent lineages [11]. It is of particular interest that rodents do not conform to Rensch´s rule, although they possess a wide range of body sizes and extensive variability in social systems. The recent analysis was based on data covering nearly 300 species, and thus the lack of conformity with Rensch´s rule could certainly not be attributed to small sample size [3]. However, it was speculated that these results may have been biased by the more extensive coverage of larger species in the dataset [3]. Moreover, the inclusion of species with different morphology, ecology and phylogenetic position into a single comparison may contribute to this pattern. Therefore, we decided to perform an analysis of SSD scaling on a new original dataset restricted to ground squirrels forming a rodent tribe that displays a wide range of body size and large diversity in social systems, yet are still similar in other aspects of their general biology.

Ground squirrels (tribe Marmotini Pocock, 1923; see [12]) are a monophyletic group within the family Sciuridae [13,14]. Ground squirrels occupy mostly open habitats of North America and Eurasia [15], are diurnal, omnivorous, reproduce usually once a year and all are semi-fossorial [15,16]. Members of the tribe display diverse social systems, from polygyny (a single male monopolizes multiple females), to promiscuity, where male success largely depends on scramble competition [17,18]. We can thus expect that the strength of sexual selection on male body size is different among particular species.

Specifically, we tested whether the allometry of SSD among ground squirrels corresponds to Rensch´s rule and whether different levels of sociality, taken as a presumable correlate of the magnitude of sexual selection on male body size, correlates with SSD and the body size pattern.

## Results

**Figure 1:**
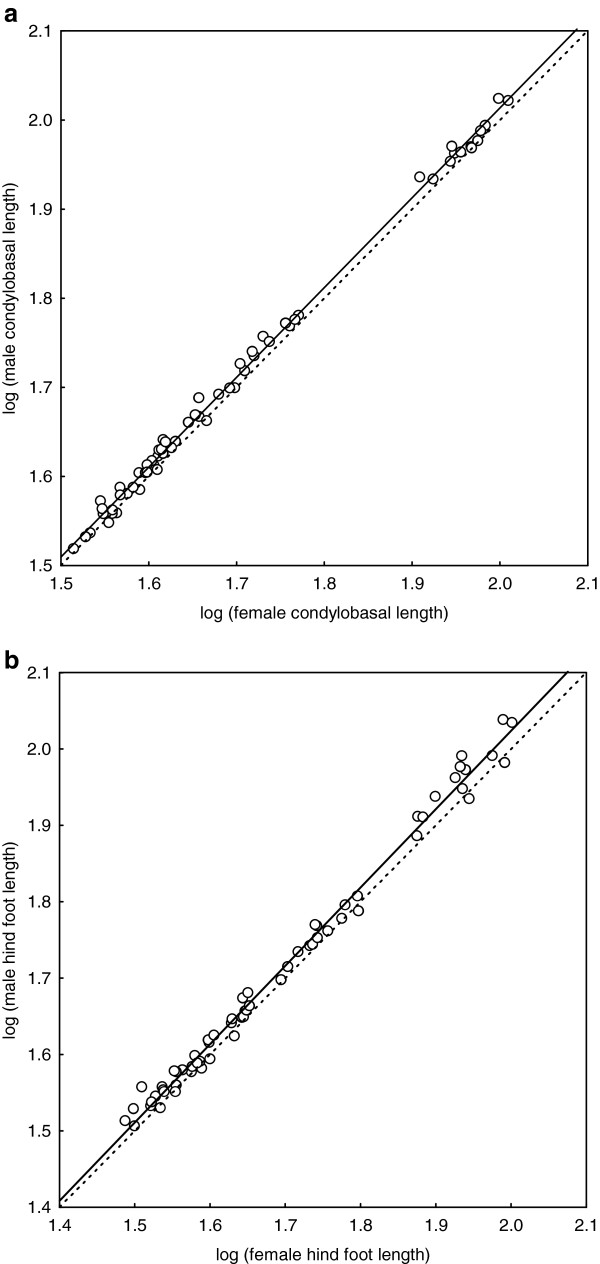
**Mean male body size increases isometrically with mean female body size among species of ground squirrels. a**) condylobasal length, **b**) hind foot length. Ordinary least-square regression (solid lines) and 1:1 relationship (dashed lines) are shown.

The two expressions of structural body size (condylo-basal length of the skull, CBL, and hind foot length, HFL) were highly correlated with each other across species (Pearson’s product–moment correlation of species means, r > 0.975, p < 0.0001, n = 63 for both males and females), but due to differences in body proportions among species, they exhibited somewhat different distributions (cf. Figure 1a, b). Although ground squirrels exhibit large variation in body size (Figure 1) and sociality, our comparison of male and female body size measurements revealed only limited variation in SSD. A significant presence of SSD among ground squirrels was found in 38% of included species based on CBL and in only 24% based on HFL (Table 1). Wherever significant, SSD proved to be male-biased, males being maximally around 8% larger in CBL and 14% larger in HFL. Estimations of SSD expressed as ratios of male to female mean CBL and HFL were correlated among species (Pearson’s product–moment correlation, r = 0.44, p < 0.001, n = 63). Both these estimations do not significantly depart from normal distribution (Kolmogorov-Smirnov tests not significant) with means 1.026 ± 0.003 (S.E.) for CBL ratio and 1.037 ± 0.005 for HFL ratio. The linear regression of log-transformed species means of male CBL on log-transformed species means of female CBL accounted for a large proportion of the variation (r^2^ = 0.997, F = 20296.75, p < 0.0001, n = 63) and showed a nearly perfectly isometric increase between male and female size across species (slope 1.008 ± 0.007 (means ± S.E. are given), 95%-CI 0.994 - 1.022; intercept n.s.; Figure 1a). Nearly identical results were found for log-transformed maximal CBL (proportion of explained variation by linear regression r^2^ = 0.994, F = 10241.77, p < 0.0001, n = 63; slope 1.014 ± 0.010, 95%-CI 0.994 - 1.034) and log-transformed mean HFL (r^2^ = 0.992, F = 7268.03, p < 0.0001, n = 63; slope 1.026 ± 0.012, 95%-CI 0.999 - 1.050; Figure 1b). In the case of log-transformed maximal HFL, linear regression (r^2^ = 0.986, F = 2105.32, p < 0.0001, n = 63) revealed a slight but significant departure from isometry (slope 1.058 ± 0.023 (S.E.), 95%-CI 1.012 - 1.105) caused by the single influential outlier, *Marmota vancouverensis*, one of the largest species. The high value of maximal HFL for males of this species was caused by a single individual with unusually long feet. We suggest that mean species values are less sensitive to such outlying individuals with extreme measurements, and we use them henceforth as more reliable expressions of size.

**Table 1:** Sociality and body size, measured as hind foot length (HFL) and condylobasal length (CBL) in males and females of 63 species of ground squirrels and the tests of sexual size dimorphism within species

**Species**	**Sociality**	**HFL**				**CBL**				**References to sociality grade**
		***n *(M,F)**	**M/F ratio**	**F**	**p**	***n *(M,F)**	**M/F ratio**	**F**	**p**	
*Ammospermophilus harrisii*	1	6,6	1.044	2.500	0.145	6,6	0.990	0.678	0.429	[19]
*Ammospermophilus insularis*	1	6,4	0.985	0.419	0.535	6,4	1.048	4.852	0.059	[20]
*Ammospermophilus interpres*	1	6,6	1.007	0.117	0.740	6,6	1.000	0.002	0.969	[21]
*Ammospermophilus leucurus*	1	6,6	1.020	0.636	0.444	6,6	1.023	2.183	0.170	[22]
*Ammospermophilus nelsoni*		6,6	1.042	4.032	0.072	6,6	1.012	1.556	0.241	
*Callospermophilus lateralis*	1	15,15	0.982	1.013	0.323	15,15	0.995	0.251	0.620	[18]
*Callospermophilus madrensis*	1	3,5	1.010	0.346	0.578	3,5	0.990	0.136	0.725	[23]
*Callospermophilus saturatus*	1	15,15	1.027	3.944	0.057	15,15	1.037	11.526	0.002**	[23]
*Cynomys gunnisoni*	4	16,14	1.024	3.416	0.075	16,14	1.036	13.928	0.001**	[18]
*Cynomys leucurus*	2	14,15	1.011	0.118	0.734	15,15	1.038	22.521	<0.001***	[18]
*Cynomys ludovicianus*	5	15,15	1.038	7.591	0.010*	15,15	1.024	7.640	0.010*	[18]
*Cynomys mexicanus*	5	5,7	0.979	0.117	0.740	5,7	1.020	2.591	0.139	[24]
*Cynomys parvidens*	4	15,10	1.064	4.136	0.054	15,10	1.064	44.093	<0.001***	[25,26]
*Ictidomys mexicanus*		9,11	1.041	2.958	0.103	9,11	1.012	0.203	0.658	
*Ictidomys parvidens*		7,7	1.048	2.101	0.173	7,7	1.036	5.986	0.031*	
*Ictidomys tridecemlineatus*	2	15,15	1.055	9.068	0.005**	15,15	1.013	1.074	0.309	[18]
*Marmota baibacina*	5	9,4	1.089	2.595	0.136	10,5	1.020	0.691	0.421	[27]
*Marmota bobac*	5	15,9	1.093	15.947	0.001**	15,11	1.033	4.604	0.042*	[27]
*Marmota broweri*	5	1,2	1.080	16.333	0.154	5,6	1.020	0.557	0.475	[27]
*Marmota caligata*	5	14,14	1.039	3.032	0.093	15,15	1.024	2.168	0.152	[27]
*Marmota camtschatica*	5	7,9	1.084	5.059	0.041*	15,17	1.024	2.018	0.166	[27]
*Marmota caudata*	5	12,8	0.978	4.136	0.054	15,15	1.023	0.974	0.332	[27]
*Marmota flaviventris*	4	22,23	1.066	10.125	0.003**	24,24	1.065	21.606	<0.001***	[17]
*Marmota himalayana*	5	4,3	1.140	5.560	0.065	14,10	1.029	2.236	0.149	[27]
*Marmota marmota*	5	12,6	0.978	0.644	0.434	17,10	1.007	0.240	0.628	[27]
*Marmota menzbieri*	5	1,2	1.028	0.870	0.522	3,3	1.023	2.638	0.180	[27]
*Marmota monax*	1	15,15	1.029	1.551	0.223	15,15	1.004	0.091	0.765	[17,27]
*Marmota olympus*	5	3,5	1.120	4.903	0.069	7,6	1.061	14.770	0.003**	[17,18,27]
*Marmota sibirica*	5	5,5	1.107	3.974	0.081	16,16	1.060	18.914	<0.001***	[27]
*Marmota vancouverensis*	5	3,4	1.081	0.358	0.576	5,4	1.002	0.008	0.931	[27]
*Notocitellus adocetus*		9,7	1.026	1.553	0.233	9,7	1.014	0.581	0.459	
*Notocitellus annulatus*		14,13	1.018	4.136	0.054	14,13	1.004	0.091	0.766	
*Otospermophilus beecheyi*	2	15,15	1.074	17.659	<0.001***	15,15	1.064	12.426	0.001*	[17]
*Otospermophilus atricapillus*		8,10	1.028	1.897	0.187	8,10	1.021	1.021	0.327	
*Otospermophilus variegatus*	3	14,15	1.028	2.446	0.129	14,15	1.022	3.951	0.057	[28]
*Poliocitellus franklinii*	1	15,15	1.056	7.736	0.010*	15,15	1.016	2.081	0.160	[18]
*Spermophilus alashanicus*		1,5	1.038	0.340	0.591	2,5	1.059	3.545	0.118	
*Spermophilus citellus*	2	15,15	1.010	0.126	0.725	17,16	1.028	3.492	0.071	J. Matějů, own data
*Spermophilus dauricus*		16,15	1.051	5.067	0.032*	16,15	1.022	2.421	0.131	
*Spermophilus erythrogenys*		15,15	1.044	5.067	0.032*	16,15	1.073	32.773	<0.001***	
*Spermophilus fulvus*	3	5,9	1.029	0.336	0.573	17,15	1.032	5.431	0.027*	[29,30]
*Spermophilus major*		15,16	1.073	4.785	0.037*	15,16	1.053	9.868	0.004**	
*Spermophilus musicus*		16,14	1.045	2.729	0.110	16,15	1.042	9.784	0.004**	
*Spermophilus pygmaeus*	2	16,15	1.073	12.634	0.001**	16,15	1.035	8.442	0.007**	[31], A.V. Tchabovsky pers. comm
*Spermophilus relictus*		6,13	0.982	0.273	0.608	6,13	0.995	0.052	0.822	
*Spermophilus suslicus*	2	16,15	1.060	7.928	0.009**	16,15	1.015	0.661	0.423	A.V. Tchabovsky pers. comm.
*Spermophilus xanthoprymnus*	2	11,11	1.061	3.615	0.072	11,11	1.046	5.715	0.027*	[32,33], V. Vohralík pers. comm.
*Urocitellus washingtoni*		15,15	1.038	4.395	0.045*	15,15	1.039	13.177	0.001**	
*Urocitellus armatus*	2	15,15	1.014	1.101	0.303	15,15	1.021	1.868	0.183	[18]
*Urocitellus beldingi*	2	15,15	1.011	0.527	0.474	15,15	1.015	2.501	0.125	[18]
*Urocitellus brunneus*		7,3	1.044	1.434	0.265	7,3	1.065	11.068	0.010*	
*Urocitellus cannus*	2	15,15	1.015	0.429	0.518	15,15	1.011	0.794	0.381	[17,18]
*Urocitellus columbianus*	3	15,15	1.008	0.208	0.652	15,15	1.029	3.526	0.071	[18]
*Urocitellus elegans*	2	15,15	1.029	2.454	0.128	15,15	1.033	7.597	0.010*	[18]
*Urocitellus mollis*	2	15,15	1.028	1.620	0.214	15,15	1.008	0.304	0.586	[17,18]
*Urocitellus parryii*	3	15,15	1.000	0.000	1.000	15,15	1.037	14.354	0.001**	[18]
*Urocitellus richardsonii*	2	15,15	1.073	20.956	<0.001***	15,15	1.036	7.284	0.012*	[18]
*Urocitellus townsendii*	2	15,12	0.993	0.629	0.472	15,12	0.986	1.658	0.210	[17,18]
*Urocitellus undulatus*		15,18	1.040	5.592	0.024*	15,18	1.038	13.657	0.001**	
*Xerospermophilus mohavensis*	1	4,2	1.036	0.629	0.472	5,6	1.008	0.757	0.407	[34]
*Xerospermophilus perotensis*		8,5	1.012	0.939	0.353	8,5	1.019	3.831	0.076	
*Xerospermophilus spilosoma*		15,15	1.030	3.811	0.061	15,15	1.027	6.995	0.013*	
*Xerospermophilus tereticaudus*	2	15,15	0.995	0.137	0.714	16,18	1.009	1.316	0.260	[18]

* p < 0.05, ** p < 0.01, *** p < 0.001.

Sociality does not significantly explain departures of mean male CBL from the common relationship with mean female CBL across species, and generally explains only a minor proportion of the total variability (ANCOVA: mean male CBL as continuous dependent variable; mean female CBL as a continuous independent variable: F_1,40_ = 5304.68, p < 0.0001, sociality coded as five grades as categorical independent variable: F_4,40_ = 2.05, p = 0.11). The results of the ANCOVA model for mean HFL as a proxy of body size were congruent with this conclusion (mean male HFL as dependent continuous variable, mean female HFL: F_1,40_ = 828.28, p < 0.0001, sociality: F_4,40_ = 0.37, p = 0.83).

**Figure 2:**
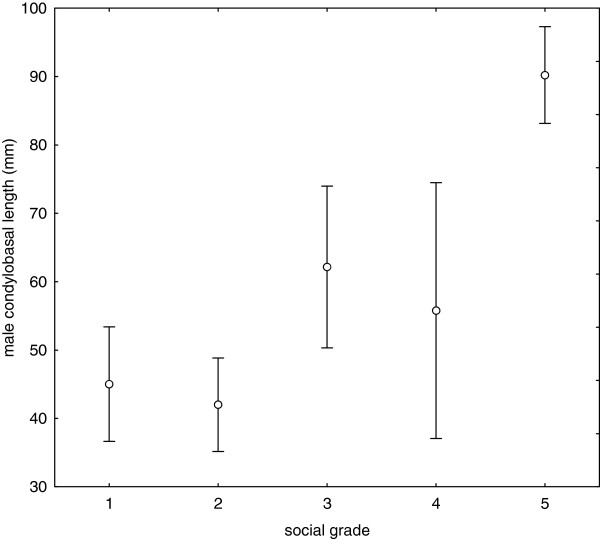
**Body size tends to increase with sociality among species of ground squirrels.** Results of one-way ANOVA of male condylobasal length results are shown as means and 95-% confidence intervals.

Nevertheless, species with different sociality grades significantly differ in body size, with the most social species tending to be the largest (one-way ANOVA of mean male CBL, factor sociality: F_4,41_ = 29.03, p < 0.0001; Figure 2; the results for female mean CBL and male and female mean HFL are practically identical). The results do not differ when sociality is coded as 0–1 dummy variable (not shown).

Phylogenetically-informed analyses confirmed that the results and their interpretations cannot be explained by a bias caused by shared ancestry. Pagel´s λ estimated in the phylogenetic generalized least squares (PGLS) regression of log-transformed mean male CBL on log-transformed mean female CBL is very low (0.07) and close to 0.0, which indicates that the effect of phylogeny on the allometry of SSD expressed in CBL is minimal. This conclusion is supported by the result of the likelihood ratio (LR) test showing that the PGLS models with λ restricted to 0.0 (equivalent to the ordinary least squares regression based on raw data) and with λ = 0.07 are statistically indistinguishable (LR_1_ = 0.32, p = 0.57). The analyses of independent contrasts, although not supported by the PGLS regression as adequate, also support the isometric increase of log-transformed male CBL with log-transformed mean female CBL (not shown). On the other hand, the effect of phylogeny is significant in the allometry of SSD based on HFL measurements, where λ estimated by maximum likelihood in the PGLS model is 0.67. The LH test confirmed significant differences between the fits of the PGLS models with λ = 0.67 and λ = 0.0 (LR_1_ = 4.06, p = 0.044). The fits of the PGLS models with λ = 0.67 and λ = 1.0, equivalent to phylogenetic independent contrasts, are not statistically different (LR_1_ = 1.68, p = 0.19). Independent contrasts in log-transformed mean male HFL and log-transformed mean female HFL scales isometrically (r^2^ = 0.960, slope 0.965 ± 0.050 is not different from 1.0 expected under isometry, n = 62 contrasts), which proves isometric scaling of male and female size in HFL as well.

Comparisons of the fits of the nested PGLS models with and without sociality as a predictor confirmed that addition of sociality into the models does not increase their explanatory power. The fit of the multivariate PGLS model of log-transformed mean male CBL on log-transformed mean female CBL and sociality coded as the five grades is not statistically different from the model after dropping of the predictor sociality (LH_1_ = 0.44, p = 0.51). The same is true for sociality coded as the 0-1dummy variable (LH_1_ = 1.44, p = 0.23). The situation for the models based on log-transformed HFL measurements is equivalent (sociality coded as five grades: LH_1_ = 0.14, p= 0.71; sociality coded as 0–1: LH_1_ = 0.02 p = 0.89). Non-significance of the factor sociality was found also in the multiple regression of independent contrasts (not shown). Estimates of Pagel´s λ were large (between 0.78 and 1.0) in all PGLS models describing association of log-transformed CBL and HFL with sociality coded either as five grades or a 0–1 dummy variable for males and females, respectively, showing a significant influence of phylogenetic relationships on the correlation between body size and sociality. The LH tests confirmed that these PGLS models are not significantly different from the respective models with λ restricted to 1.0 equivalent to phylogenetic independent contrasts (all p > 0.15). The analyses of phylogenetic contrasts confirmed significant correlations between log-transformed mean CBL and mean HFL in males and females and sociality coded as five grades and 0–1, respectively (all r > 0.39, p < 0.05, n = 45 contrasts).

Exclusion of seven species (*Ammospermophilus insularis, Callospermophilus madrensis, Marmota menzbieri, Marmota vancouverensis, Urocitellus brunneus* and *Spermophilus alashanicus*) with sample size less than five individuals for at least one sex in CBL does not change significance of any results and the major interpretations are thus robust with respect to inclusion of these species with small sample size.

## Discussion

Our results revealed only limited variation in SSD in two measurements representing different aspects of structural body size among ground squirrels, although we stress that the lack of significant SSD in some species can be attributed to small sample size. This variability in SSD cannot be explained by either of the two commonly reported correlates of SSD, i.e. body size and social system. Ground squirrels thus do not conform to Rensch’s rule, in contrast to the general trend in mammals [3]. Our results, based on an original morphometric dataset covering most species in a monophyletic group with similar general biology, are thus in line with a previous report showing similar results in rodents [3]. Rensch´s rule is usually not followed in groups with female-biased SSD [35-37]. Here, we report that it is not followed in a mammalian group with predominantly male-biased SSD.

We suggest that the limited extent of SSD in structural body size and the lack of the support for Rensch´s rule in ground squirrels could be generally explained by three possible scenarios: i) low selection on SSD in structural dimensions, ii) ontogenetic and genetic constraints or iii) the existence of ecological/selection factors preventing the evolution of extensive SSD.

Male-biased SSD, typical for mammals, is usually associated with sexual selection on body size enlargement that correlates with success in female monopolization. Reiss [38] argued that the relationship between body size and SSD found in some mammalian lineages can be largely explained by the co-variation of mating systems with body size: larger species have a larger opportunity for polygyny and thus stronger selection for male size increases leading to male-biased SSD. The pattern consistent with Rensch´s rule driven probably by sexual selection in males has also been found in other animal groups, i.e. shorebirds [39]. In ground squirrels, social organization changes with body size. Small species are typically less social and hence presumably more promiscuous [18], while the more social larger species probably exhibit larger selective pressure on female monopolization and hence body size enlargement in males. This is also evident in marmots, the largest species of ground squirrels, where polygyny or facultative monogamy is based on active female-defence [18]. A correspondence between social grade and sexual selection on male size enlargement in ground squirrels is supported by single-species studies. For instance, success in male-male combat over females is positively related to male body size in *Urocitellus parryii*[40], where territorial males are associated with female kin clusters (social grade 3; [18]). Similarly, larger males of most *Cynomys* species (grades 3–5) are more successful in securing breeding territories and sire more offspring [41]. On the other hand, selection pressure towards smaller male body size was observed in the low social (grade 2) promiscuous *Ictidomys tridecemlineatus*, where males are selected for maneuverability and early sexual maturation [42].

In summary, selection for male body size enlargement should increase with increased sociality and body size in ground squirrels, which should lead to a pattern consistent with Rench’s rule. We can thus tentatively conclude that differences in sexual selection among ground squirrels, although associated with body size variation, do not lead to conformity with Rensch´s rule. The support for Rensch´s rule across breeds of domestic mammals suggests that the allometry for SSD could be a more general consequence of body size evolution caused by different selective agents [43,44]. Nevertheless, ground squirrels do not follow the rule although they have large variation in body size. We suggest that this state can be explained by the limited extent of SSD in this rodent group.

Limited SSD could reflect ontogenetic or genetic constraints, an idea going back to Charles R. Darwin [1], see also e.g. [45,46]. It is notable that a generally low degree of SSD in structural body size has been reported for rodents [11], although they inhabit a wide variety of habitats and possess various social systems. Nevertheless, it is not clear why rodents should have, for instance, a stronger intersexual genetic correlation in body size than other animal groups. Moreover, the existence of SSD (although with limited magnitude) in some species of ground squirrels suggest that there is sufficient genetic variation for SSD and that this trait should thus be subject to evolution.

Alternatively, selection may operate on male and female structural size independently, males being shaped by sexual selection and females by fecundity selection, but the optimal size for both sexes can be similar. In most ground squirrel species, female reproductive success (litter size, progeny survival) is positively related to body size [41,47-49]. Selection pressure towards larger female body size may contribute to low SSD in ground squirrels; however, it is not clear why male and females of species differing in body size and thus the energetics of growth and reproduction, and also having different mating or social systems, should always have a similar optimal body size.

It is also possible that there is selection for an increase of SSD in structural size, but that this pressure is counterbalanced by other selective pressures constraining its extent and resulting in both sexes having a similar, optimal structural body size. One potential limiting factor for large differences in such SSD is the subterranean environment. Both male and female ground squirrels occupy underground burrows, which they often share for mating, shelter e.g. [50,51] and even hibernation in some marmot species [16,50]. The disadvantage of significant male and female body size differences in this case is apparent – large animals can suffer locomotion difficulties in tunnels made by smaller individuals. This hypothesis makes a straightforward prediction that SSD should be larger in species where both sexes do not share common underground tunnels, which could be tested in future comparative analyses.

It is necessary to stress that the limited extent of SSD in ground squirrels may only be restricted to structural size measurements. Sexual differences in ground squirrel body mass are often more pronounced (for reviews see [11,52]). For example, seasonally variable sexual body mass dimorphism was found in Utah prairie dogs (*Cynomys parvidens*) where the M:F ratio varies across seasons between 0.99 and even 1.53 [41]. Part of this large range in body mass SSD can be attributed to the fact that body mass increases approximately with the third power of length measurements. Nevertheless, the SSD index for mean CBL in this species is only around 1.06, which predicts that males should be only 20% heavier than females. It is evident that SSD in body mass reflects not only differences in structural body size, but also different allocations of males and females to fat reserves or musculature and different energetic consequences of reproduction.

Although SSD in structural body size is not associated with body size and sociality, sociality and body size proved to be highly correlated (Figure 2). Solitary and gregarious species (grades 1 and 2) tend to be small, while especially polygynous and/or monogamous species living in stable family groups (grade 5) are large. The ultimate explanations of the evolution of social living in ground squirrels may be anti-predator behaviour and the male protection of offspring against infanticide [18]. Correlation between sociality and body size in ground squirrels can be proximately explained by longer postnatal growth and development in large species, which leads to the longer retention of offspring within the maternal home range [17] and a large coincidence of above-ground activity between adult and immature cohorts [18], hence allowing a longer time for the building of social contacts.

## Conclusions

The analyses of our original dataset confirmed low SSD in the structural dimensions of ground squirrels, a group with variable social systems and large variation in body size. We also found that male and female body structural sizes increase nearly perfectly isometrically, and that the group does not follow the otherwise widely applicable Rensch´s rule. Negative results are more difficult to explain than positive findings; however, we suggest that the lack of conformity with Rensch´s rule in ground squirrels may be attributed to the generally low variation of SSD in structural size in rodents, a phenomenon that deserves further comparative work.

## Material and methods

We measured CBL in 1527 specimens from museum collections. Our material covers 63 out of 68 species of ground squirrels recently recognized in the tribe Marmotini sensu [12]; for the number of species see [53]. Only adult, undamaged and located specimens (modus 15 per sex-species category) were examined. Adults were identified according to the degree of teeth abrasion or stage (adult/juvenile) was obtained directly from specimen tags. Our dataset can be biased by geographic intraspecific variability in body size documented in ground squirrels [54]. Nevertheless, taking into account the large variability in body size across species of the studied group, we expect that intraspecific variability does not largely influence the interspecific pattern. Moreover, only individuals determined as a same subspecies and/or collected within a restricted geographic area were measured in most species. Four *Spermophilus* species (*S. ralli, S. pallidicauda, S. taurensis* and *S. brevicauda*) and *Marmota kastschenkoi* were not included into our study, as we were not able to obtain enough specimens. All CBL measurements were taken by a single person (JM). Data on HFL for adult males and females of each species were taken from museum tags (in total available in 1392 specimens, Table 1).

Body mass is often used as a measure of body size in the SSD literature, including [3]. Nevertheless, body mass is not a good expression of body size in ground squirrels [54], because many species considerably fluctuate in body mass throughout the year. For instance, data on body mass before and after hibernation are highly different e.g. [55] and are not comparable to body mass in less seasonal species. Our measurements, CBL and HFL, represent the structural component of size that is less dependent on body conditions. Moreover, it is known that HFL in rodents is among those external measurements that reach their final size very early during postnatal ontogeny e.g. [56,57]. There is not a single perfect measurement expressing general body size (see e.g. [58]) and most body measurements use to be highly intercorrelated in morphometric analyses. For example, skull measurements are highly correlated with each other in ground squirrels [54]. To avoid this problem, we selected CBL and HFL, because they reflect size of very different body parts, although due to large variability in body size among ground squirrels, we can *a priori* expect that these measures will be highly correlated across all species included (we tested the correlation by Pearson test for males and females separately).

The data on social organization were obtained in two ways. Most of information was taken from literature concerning ground squirrel sociality [17,18,27]. Second, the level of sociality was classified using publications on general biology (see Table 1 for particular references) or our personal knowledge of the species biology (*S. citellus*). In this way, sociality was particularly assessed for the genus *Ammospermophilus*, species *Callospermophilus madrensis, C. saturatus, Cynomys mexicanus, C. parvidens, Otospermophilus variegatus, Spermophilus citellus, S. suslicus, S. pygmaeus, S. fulvus*, *S. xanthoprymnus* and *Xerospermophilus mohavensis.* Moreover, in the case of mentioned Palaearctic species, the social grades assignment was consulted with A. V. Tchabovski (N. A. Severtsov Institute of Ecology and Evolution, Russian Academy of Sciences) and V. Vohralík (Faculty of Science, Charles University in Prague). In classifying the species social grades, we especially consider descriptions of inter-individual contacts, age of first reproduction, age of dispersal, character of dispersal and density of individuals (see [17] documenting correlation of sociality and the mentioned variables).

Species were classified into five categories representing different levels of sociality defined by [18]: 1 – asocial, 2 – single-family female kin clusters, 3 – female kin clusters with a territorial male, 4 – polygynous harems with male dominance, 5 – egalitarian polygynous harems. Facultative monogamy, known in some marmots, was included in the last category, as it is usually interpreted as an extreme case of polygyny where males are unable to monopolize more than one female under harsh environmental conditions ([59] and references therein). In cases of transient classification (i.e. level 1 – 2 in *Urocitellus townsendii*, *Ictidomys tridecemlineatus*, and 2 – 3 in *Otospermophilus beecheyii*) of species sociality in [18], we adopted the social grades of [17]. Despite extensive literature search we were not able to obtain or estimate data about sociality in 17 species from our morphological dataset. These species are thus not included into the analyses concerning effects of sociality. The sociality scale in ground squirrels forms a continuum from the least social (grade 1) to the most social (grade 5) organization; species placed into same category have similar but not always identical social system [18]. Throughout this work, we assume that these social grades reflect the strength of sexual selection on males. However, we are aware that social systems are not always necessary surrogates for mating systems, e.g. [60]. We summarized information about species mating systems as well, but primary data for the group are scarce and frequently in contradiction, for a review see [59]. Moreover, the definition of the polygynous mating system is disputable in rodents [59].

The significance of SSD in a given species was tested by one-way ANOVAs. We expressed SSD as ratios of male to female mean CBL and HFL and tested whether the SSD estimation from CBL and HFL are correlated across species by Pearson´s product–moment correlation. We also tested whether these SSD indexes follow normal distribution across species by Kolmogorov-Smirnov test. Ordinary least square regression of log-transformed mean or maximal size measurements in males on log-transformed mean or maximal size measurements in females was used for testing the allometry of SSD and hence Rensch´s rule. Slope 1.0 was expected under isometric increase of male size with female size. The ANCOVA models with size measurements in males as the dependent continuous variable, size measurements in females as the continuous independent variable and degree of sociality as the independent categorical variable were used to test the relationship between SSD and sociality among species. We tested the effect of sociality on species body size by one-way ANOVAs with male, respectively female body size measurements as dependent variable and sociality as factor.

Because species data are not independent, we performed analyses that take the phylogenetic relationship among species into account as well, specifically, the analyses of phylogenetic independent contrasts and PGLS [61-63]. We used the topology of the tribe published recently by [14]. Three species (*U. cannus, U. mollis* and *A. nelsoni*) are missing in their tree. We took the position of *U. cannus* and *U. mollis* from [13]. *A. nelsoni* is considered to diverged from *A. leucurus*, following [64], we took it as sister to this species. See the Additional file 1 for the whole composite tree. The branch length estimations for our tree are not available. Nevertheless, simulated studies showed that the independent contrasts method is sufficiently robust to errors in branch lengths [65]. We report results based on arbitrary, equal branch length both in PGLS models and in analyses of phylogenetic independent contrasts. In some cases, the phylogenetic contrasts based on equal branch length did not meet assumptions suggested by [66]. Therefore, in the analyses of independent contrasts, we used Graafen´s branch lengths instead as well, which did not lead to significant correlations between contrasts and branch length [66]. The results of all analyses of phylogenetic independent contrasts computed using either equal or Graafen´s branch lengths were equivalent; therefore, we report only the results for equal branch length.

We used phylogenetic independent contrasts [62] and PGLS models [63,67] - for recent application, discussion and minute description of the PGLS technique see e.g. [68,69] - to test for allometry in SSD, association of SSD with sociality and correlation between body size and sociality. In PGLS, the λ parameter is found by maximum likelihood. This parameter potentially varies between 0, indicating no effect of phylogenetic signal, and 1, corresponding to the analysis of independent contrasts where trait variation among species is predicted by phylogeny. Fit of nested PGLS models (models with and without a particular predictor) or PGLS models with and without a parameter restricted to a constant (e.g. λ can be restricted to 0 or 1, estimation of the λ parameter requires 1 degree of freedom) can be compared using a LR test: LR_df_ = −2 × [Lh (better-fitting model) – Lh (worse-fitting model)], where the best fitting model has the highest log-likelihood (Lh) score. The significance of this difference can be evaluated with a *χ*^2^ distribution with degrees of freedom equal to the difference in the number of parameters between the two competing models [63]. We used this approach for comparison of PGLS models with λ estimated by maximum likelihood with the analyses of raw data (λ restricted to 0 in PGLS) or of independent contrasts (λ restricted to 1) to estimate the impact of the phylogenetic signal for a given analysis. Further, we applied this procedure for comparison of PGLS models with and without predictor sociality to test the association of sociality on SSD.

Degree of sociality is an ordered variable. In many cases, an ordered variable represent coarse information about an underlying continuous variable, which is also true in our case [18]. Thus, we used it in PGLS regressions and analysis of independent contrasts as a continuous variable. This approach may lead to increased Type I error largely due to potentially unequal distances between subsequent categories [70]. To control for this potential bias, we also coded sociality as a variable with two levels, that can be used as a dummy variable in regressions. In this approach, we coded sociality grades 1 and 2 as 0, and the grades 3, 4 and 5 as 1. This division is based on the expectation that different social organization should be associated with different potential for female monopolization, which should be connected with the strength of major selective pressure for male body size enlargement. And just the social grades 1–2 and 3–5 differ in the presence of the stable association of males with females.

Because sample size was small for some species, we repeated all analyses after exclusion of species with the sample size in CBL less than 5 for a minority sex.

We used an α level of significance of 0.05.

All analyses were performed in Statistica 10.0 (Stat Soft 2011), the PDAP:PDTREE module [71] within Mesquite 2.75 [72] and Compare vers. 4.6b [73] for independent contrasts and BayesTraits [74] for PGLS models.

Ethical note: The study is based on measurements of museum specimens and does not involve living animals.

## Competing interests

The authors declare that they have no competing interests.

## Authors’ contributions

JM and LK conceived of, designed and coordinated the study and drafted the manuscript. JM collected the data and LK performed the statistical analyses. Both authors read and approved the final manuscript.

## Supplementary Material

Additional file 1 The composite tree of ground squirrels used for the phylogenetic comparative part of the study. Click here for file
