# Supplementary figures and images for: Sexual size dimorphism in ground squirrels (Rodentia: Sciuridae: Marmotini) does not correlate with body size and sociality
Source: Front Zool. 2013 May 14;10:27. doi: 10.1186/1742-9994-10-27 (PMC3663772; doi:10.1186/1742-9994-10-27)

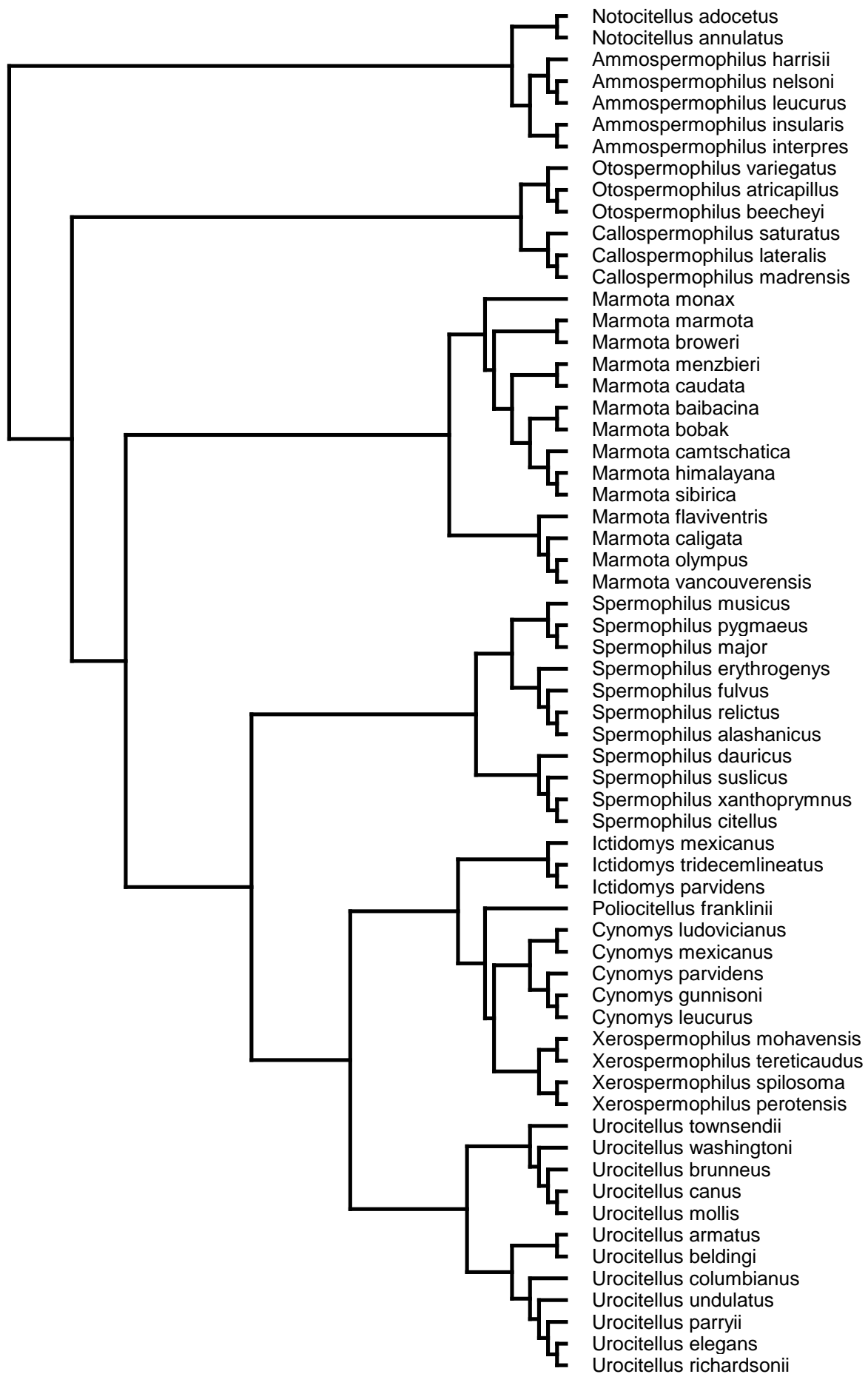

Supplement: Additional file 1 — The composite tree of ground squirrels used for the phylogenetic comparative part of the study. [file 1742-9994-10-27-S1.pdf]
